# Mohs micrographic surgery versus wide local excision for recurrent cutaneous squamous cell carcinoma

**DOI:** 10.1016/j.jdin.2026.05.013

**Published:** 2026-05-23

**Authors:** Nina A. Ran, Cristian Cardona-Machado, Emily E. Granger, David G. Brodland, David R. Carr, Joi B. Carter, John A. Carucci, Kelsey E. Hirotsu, Shlomo A. Koyfman, Aaron R. Mangold, Fabio Muradás Girardi, Kathryn T. Shahwan, Divya Srivastava, Rajiv I. Nijhawan, Allison T. Vidimos, Tyler J. Willenbrink, Ashley Wysong, Emily S. Ruiz, Javier Cañueto

**Affiliations:** a Department of Dermatology, Brigham and Women’s Hospital, Boston, Massachusetts; b Department of Dermatology, Complejo Asistencial Universitario de Salamanca, Salamanca Spain; c Department of Medicine, Newton-Wellesley Hospital, Boston, Massachusetts; d Zitelli and Brodland P.C., Pittsburgh Pennsylvania; e Department of Dermatology, The Ohio State University Medical Center, Columbus Ohio; f Department of Dermatology, Geisel School of Medicine at Dartmouth, Hanover New Hampshire; g Department of Dermatology, New York University School of Medicine, New York New York; h Stanford School of Medicine, Stanford California; i Department of Radiation Oncology, Cleveland Clinic, Cleveland Ohio; j Department of Dermatology, Mayo Clinic Arizona, Scottsdale Arizona; k Department of Head and Neck Surgery, Ana Nery Hospital, Santa Cruz do Sul, RS, Brazil; l Department of Dermatology, University of Texas Southwestern School of Medicine, Dallas Texas; m Department of Dermatology, Cleveland Clinic, Cleveland Ohio; n Department of Dermatology, University of Nebraska Medical Center, Omaha, Nebraska; o Instituto de Investigación Biomédica de Salamanca (IBSAL), Salamanca, Spain; p Faculty of Medicine, University of Salamanca, Salamanca, Spain

**Keywords:** cutaneous squamous cell carcinoma, dermatologic surgery, epidemiology, Mohs micrographic surgery, recurrent squamous cell carcinoma

*To the Editor:* Cutaneous squamous cell carcinomas (CSCC) that present as locally recurrent tumors (recurrent CSCC, or rCSCC) have higher rates of local recurrence (LR), regional metastasis (RM), and disease-specific death (DSD) after surgery.^1^ Recent data indicate that Mohs micrographic surgery (MMS) provides superior outcomes compared with wide local excision (WLE) for primary high-stage CSCC; however, the 2 surgical approaches have not been compared for rCSCC.^2^^,^^3^ This study uses a large retrospective multicenter cohort to evaluate whether MMS is associated with reduced risks of poor outcomes compared with WLE in rCSCC.

Data were derived from 12 centers in the United States, Spain, and Brazil, and the methodology for aggregating this data set was recently described.^4^ This study included only rCSCC, defined as a tumor with comparable histology arising within the area of the previously treated tumor.^5^ All rCSCC were resected with MMS or WLE with negative surgical margins. Tumors were excluded if they were metastatic at presentation, received neoadjuvant or adjuvant therapy, or had unknown follow-up. This study evaluated 3 outcomes: (1) LR; (2) RM, defined as the presence of in-transit or nodal metastasis; and (3) DSD, defined as death from CSCC or its treatment.

The statistical methodology is described in detail in the Supplemental Materials, (available via Mendeley at https://data.mendeley.com/drafts/pdxcjx2rf3). Because CSCC undergoing MMS versus WLE have different baseline characteristics (confounding by indication), propensity-score-based weighting was performed to balance baseline patient and tumor characteristics before outcomes modeling. Any characteristic not balanced by propensity-score-based weighting was included as a covariate in the outcomes modeling (double robust estimation method). Weighted fine-gray competing-risk regression was performed to derive subdistribution hazard ratios and compute 5-year cumulative incidence for each outcome. Given the imbalance in perineural invasion (PNI) between treatment groups, a sensitivity analysis excluding tumors with PNI was performed.

A total 265 rCSCC were included, of which 219 (82.6%) were treated with MMS (Table I). MMS-treated rCSCC were more likely to be from male patients (78.5% vs 63.0% for WLE), to be located on the head/neck (79.0% vs 54.3%), and to have a diameter at least 2cm (71.7% vs 39.1%) and deep invasion (75.3% vs 19.6%). WLE-treated rCSCC were more likely to be from immunosuppressed patients (32.6% vs 18.3%) and to have PNI (15.2% vs 0.9%). In total, the cohort experienced 21LR (MMS:16; WLE:5), 34RM (MMS:29; WLE:5), and 13DSD (MMS:6; WLE:7).

**Table I:** Patient demographics, tumor characteristics, treatment, and outcomes for primary and recurrent cutaneous squamous cell carcinomas

Variables	Recurrent cohort			Matched cohort		
	Excision *n* = 46	Mohs *n* = 219	SMD	Excision *n* = 45.79	Mohs *n* = 215.93	SMD
Follow-up, mo, median [IQR]	25.0 (16.8-47.7)	48.5 (25-87)	0.551	28.10 (9-29)	53.73 (24-84)	0.768
Age (y), mean ± SD	75.46 ± 11.33	76.10 ± 11.22	0.056	76.56 ± 11.59	76.12 ± 11.15	0.039
Sex, *n* (%)			0.346			0.048
Male	29 (63.0)	172 (78.5)		34.0 (77.4)	163.4 (75.4)	
Female	17 (37.0)	47 (21.5)		9.9 (22.6)	53.5 (24.6)	
Immune status, *n* (%)			0.334			0.184
Immunocompetent	31 (67.4)	179 (81.7)		31.0 (70.5)	170.2 (78.5)	
Immunosuppressed	15 (32.6)	40 (18.3)		13.0 (29.5)	46.7 (21.5)	
Anatomic location, *n* (%)			0.542			0.046
Head/Neck	25 (54.3)	173 (79.0)		12.2 (27.8)	55.9 (25.8)	
Trunk/extremities	21 (45.7)	46 (21.0)		31.8 (72.2)	160.9 (74.2)	
Preoperative diameter, *n* (%)			0.693			0.020
Diameter <2 cm	28 (60.9)	62 (28.3)		14.6 (33.2)	74.1 (34.2)	
Diameter at least 2 cm	18 (39.1)	157 (71.7)		29.4 (66.8)	142.7 (65.8)	
Depth of invasion, *n* (%)			1.347			1.510
Dermis/subcutis	37 (80.4)	54 (24.7)		39.6 (90.0)	67.5 (31.1)	
Deep invasion∗	9 (19.6)	165 (75.3)		4.4 (10.0)	149.3 (68.9)	
Histologic differentiation, *n* (%)			0.100			0.084
Well/moderate	35 (76.1)	157 (71.7)		30.7 (69.9)	159.7 (73.7)	
Poor	11 (23.9)	62 (28.3)		13.3 (30.1)	57.1 (26.3)	
Perineural invasion, *n* (%)	7 (15.2)	2 (0.9)	0.544	1.6 (3.5)	5.8 (2.7)	0.048
Lymphovascular invasion, *n* (%)	3 (6.5)	0 (0.0)	0.374	0.5 (1.2)	0.0 (0.0)	0.155
BWH stage			1.135			0.660
T1	21 (45.7)	31 (14.2)		10.1 (23.0)	42.4 (19.6)	
T2a	9 (19.6)	35 (16.0)		16.9 (38.4)	35.3 (16.3)	
T2b	11 (23.9)	152 (69.4)		15.5 (35.3)	138.2 (63.7)	
T3	5 (10.9)	1 (0.5)		1.4 (3.3)	0.9 (0.4)	
AJCC8 stage			0.880			0.389
T1	22 (47.8)	33 (15.1)		10.3 (23.4)	44.2 (20.4)	
T2	4 (8.7)	17 (7.8)		7.8 (17.8)	15.7 (7.2)	
T3	18 (39.1)	168 (76.7)		25.1 (57.0)	156.1 (72.0)	
T4a	2 (4.3)	1 (0.5)		0.8 (1.8)	0.9 (0.4)	

*AJCC8*, American Joint Committee on Cancer 8th Edition; *BWH*, Brigham and Women’s Hospital; *MMS*, Mohs micrographic surgery; *SD,* standard deviation; *SMD*, standardized mean difference; *WLE*, wide local excision.

∗ Deep invasion defined as invasion beyond subcutaneous fat or depth at least 6 mm.

Propensity score-based weighting balanced all baseline characteristics except immune status, depth of invasion, and lymphovascular invasion, which were included in the regression modeling. On multivariable regression, MMS was associated with a decreased risk of LR (SHR = 0.07 [0.02-0.33]; *P* = .001) and DSD (SHR = 0.13 [0.03-0.51]; *P* = .003) compared with WLE. No difference was observed for RM. MMS-treated rCSCC had a lower estimated 5-year cumulative incidence of LR (1.1% vs 13.9% for WLE) and DSD (0.15% vs 1.17% for WLE; Fig 1). Results were consistent in a sensitivity analysis excluding tumors with PNI (Supplementary Table I, available via Mendeley at https://data.mendeley.com/drafts/pdxcjx2rf3).

**Fig 1:**
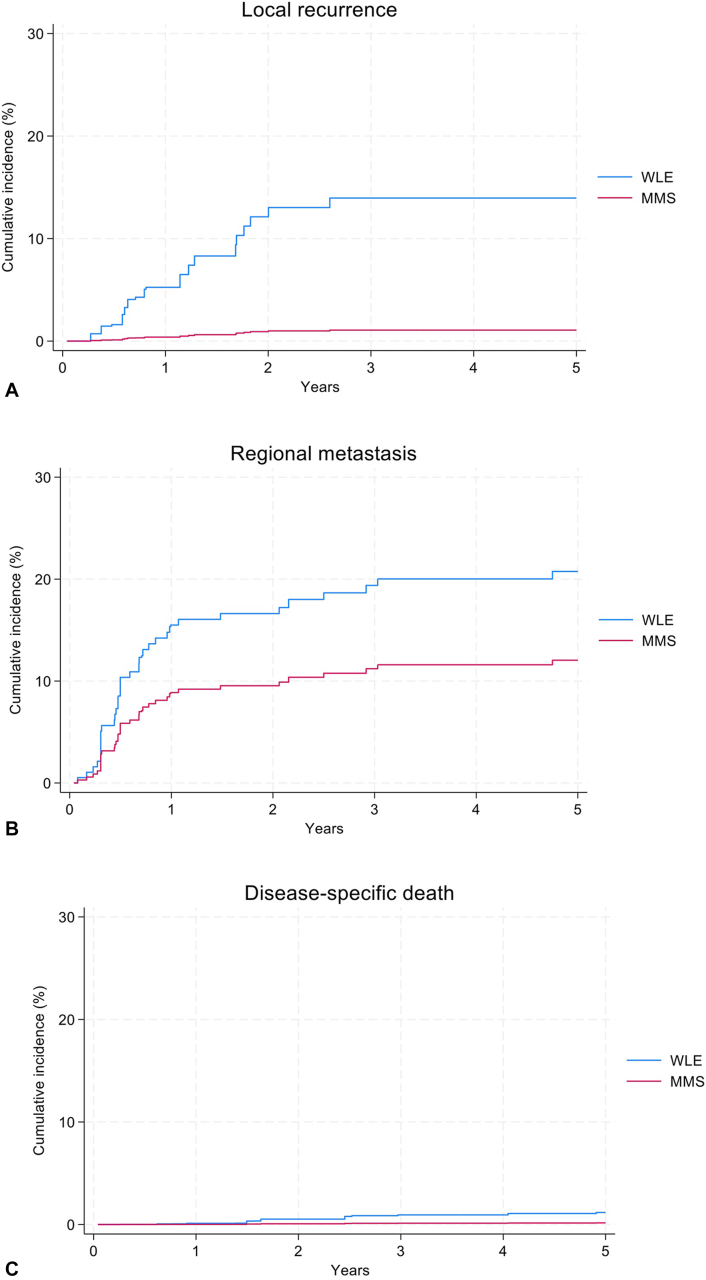
Five-year cumulative incidence of local recurrence **(A)**, regional metastasis **(B)**, and disease-specific death **(C)** for recurrent squamous cell carcinomas treated with wide local excision versus Mohs micrographic surgery.

This study has some limitations. The number of DSD was limited; the exclusion of patients treated with adjuvant radiotherapy may not fully reflect the real-life setting; detailed margin information in the WLE cohort was not available. Beyond these limitations, in this retrospective multicenter cohort of 265 rCSCCS, MMS was associated with a lower observed risk of LR and DSD.

## Conflicts of interest

Dr Cañueto has the following disclosures: grants to institution: Sanofi/Regeneron, Castle Biosciences; Lectures honoraria: Sanofi, Almirall, AbbVie, Regeneron, Sun Pharma; Payment for expert testimony: Sanofi, Regeneron, Almirall; Support for attending meetings: Pfizer, Almirall, Lilly, Castle Biosciences; Advisory board: Almirall, Sanofi, Regeneron, Kyowa, Roche, and InflaRx. Dr Ruiz serves is a consultant for Regeneron, Checkpoint Therapeutic, Feldan Pharmaceuticals. She serves as a Principal Investigator/Co-Investigator for the following companies: Regeneron (PI/Co-I), Merck (Co-I), Castle Biosciences (Co-I) and is the Executive Officer of Skin Cancer Outcomes Consortium (SCOUT) Skin Cancer Champions. Dr Carr is an investigator for Regeneron (no direct funds). Dr Mangold has consulted for Phelecs BV, Kyowa, Eli Lilly, Momenta, UCB, and Regeneron in the past, greater than 24 months ago. He has consulted for Incyte, Soligenix, Clarivate, Argenyx, and Bristol Myers Squibb in the past, less than 12 months ago. He consults for Nuvig, Tourmaline Bio, Janssen, Boehringer Ingelheim currently. He consults for Regeneron and Pfizer currently with payments to the institution. He has grant support from Kyowa, Miragen, Regeneron, Corbus, Pfizer, Incyte, Eli Lilly, Argenx, Palvella, AbbVie, Priovant, Merck in the last 24 months. Beyond 24 months, grant support has come from Sun Pharma, Elorac, Novartis, and Janssen. He has received royalties from Adelphi Values and Clarivate. His current patents include Methods and Materials for assessing and treating cutaneous squamous cell carcinoma (provisional PCT/US2023/078902), Use of Oral Jaki in Lichen Planus- PCT/US2024/020149; and Topical Ruxolitinib in Lichen Planus- PCT/US2021/053149, 2023-520085, & 21805700.8, respectively. Dr Carucci receives funding for investigator initiated basic science research from Regeneron and is a PI for a clinical trial sponsored by Regeneron. Dr Koyfman has the following disclosures: Advisory board (paid): Merck, BMS, Regeneron, Galera therapeutics; Advisory board (unpaid): Castle Biosciences; Research support: Castle biosciences, Merck, BMS, Regeneron; Honoraria: UpToDate. Dr Vidimos has the following disclosures: Research Support – Castle**;** Advisory Board - Inhibitor Therapeutics; Honoraria - Up to Date. Dr Wysong has received an institutional research grant from Castle Biosciences. Dr Ran serves as a consultant for Chronicle Medical Software. Drs Granger, Shahwan, Hirotsu, Carter, Girardi, Brodland, Srivastava, Cardona-Machado, Nijhawan, and Willenbrink have no conflicts of interest to declare.
